# Translating Cochrane Reviews to Ensure that Healthcare Decision-Making is Informed by High-Quality Research Evidence

**DOI:** 10.1371/journal.pmed.1001516

**Published:** 2013-09-17

**Authors:** Erik von Elm, Philippe Ravaud, Harriet MacLehose, Lawrence Mbuagbaw, Paul Garner, Juliane Ried, Xavier Bonfill

**Affiliations:** 1 Cochrane Switzerland, Institute of Social and Preventive Medicine (IUMSP), Lausanne University Hospital, Lausanne, Switzerland; 2 French Cochrane Centre; Assistance Publique-Hôpitaux de Paris, Hôpital Hôtel-Dieu, Centre d'Epidémiologie Clinique; Université Paris Descartes - Sorbonne Paris Cité, Paris, France; INSERM U738, Paris, France; 3 Cochrane Editorial Unit, London, United Kingdom; 4 Department of Clinical Epidemiology and Biostatistics, McMaster University, Hamilton, Ontario, Canada; 5 Centre for Development of Best Practices in Health, Yaoundé Central Hospital, Yaoundé, Cameroon; 6 Department of Clinical Sciences, Liverpool School of Tropical Medicine, Liverpool, United Kingdom; 7 The Cochrane Collaboration, Oxford, United Kingdom; 8 Iberoamerican Cochrane Centre, Institute of Biomedical Research (IIB Sant Pau), CIBER de Epidemiología y Salud Pública (CIBERESP), Universitat Autònoma de Barcelona, Barcelona, Spain

## Abstract

Erik von Elm and colleagues discuss plans to increase access and global reach of Cochrane Reviews through translations into other languages.

*Please see later in the article for the Editors' Summary*

**Summary Points**

- Cochrane Reviews, systematic reviews prepared by The Cochrane Collaboration, aim to inform healthcare decision-making anywhere in the world by providing high-quality timely critical summaries of research evidence.
- All Cochrane Reviews are prepared and published in English, but during its 20^th^ anniversary year, The Cochrane Collaboration is responding to the challenge to increase access and global reach through translations into other languages.
- Current projects to translate Cochrane content into Spanish and French are promising as usage statistics increase with greater provision of translated content. Enhanced ways to search and access Cochrane Reviews in different languages will improve the user experience and availability of content.
- New technologies, such as machine translation using learning systems, translation crowd-sourcing, and the use of a controlled language for the original English version have the potential to considerably improve possibilities to translate Cochrane content at large scale and in several languages.

The vision of The Cochrane Collaboration is that healthcare decision-making be informed by high-quality, timely research evidence. Now, 20 years on from when it was established, the organization is making a substantive contribution globally to realizing this vision. Systematic reviews prepared and maintained by members of The Cochrane Collaboration are published in the *Cochrane Database of Systematic Reviews* (*CDSR*). As one of the seven databases in *The Cochrane Library* (www.thecochranelibrary.com), it includes all completed Cochrane Reviews and Protocols outlining the methods of reviews in progress. Both Protocols and full versions of the Reviews follow rigorous methodology and are peer-reviewed. They differ from other (narrative) types of literature reviews that are common in the medical literature (Table S1) but share features of good quality with many systematic reviews published elsewhere. Launched in 1995, the *CDSR* evolved from previously existing databases [1]. The first issue included 36 Cochrane Reviews and 16 Protocols. The *CDSR* has grown steadily to more than 5000 full Reviews and over 2000 Protocols across all areas of healthcare, most focusing on the effects of interventions. In parallel, global access to the *CDSR* has more than doubled since 2006 (Figure 1).

**Figure 1:**
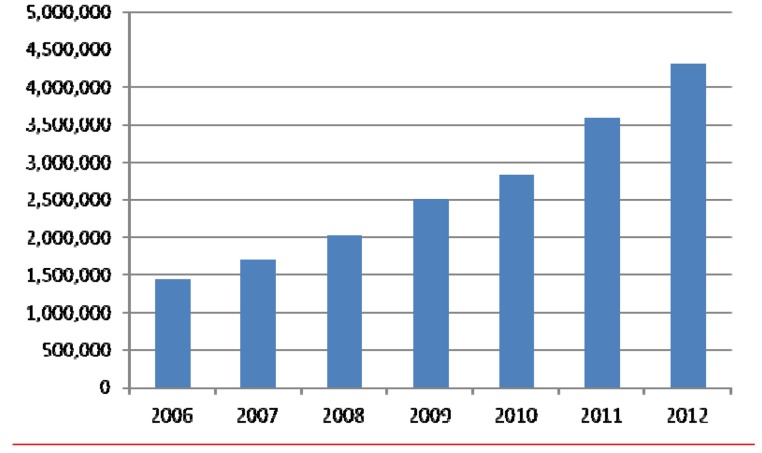
Full-text downloads of Cochrane Reviews, 2006 to 2012.

Over the past two decades, The Cochrane Collaboration has broadened the international reach of its membership: from some 77 people from nine countries, who met for the first Cochrane Colloquium in 1993, to more than 28,000 contributors in over 100 countries in 2013. This international growth did not happen by chance: The Cochrane Collaboration's strategy from the start has been to train people for evidence synthesis and to build research capacity, principally through Cochrane Centres and Branches located in Europe, the Americas, Asia, Africa, and Australasia.

Cochrane Reviews are prepared and published in English as many people in the organisation are from English-speaking countries and because English dominates scientific communication. Currently, however, The Cochrane Collaboration is examining options for multilingual publication of Cochrane Reviews and, in coincidence with its 20^th^anniversary, has made strategic decisions to address this issue. This Essay describes how the organisation is responding to the challenge of promoting evidence-informed health care by publishing its high-quality content in languages other than English.

## Translations of Cochrane Reviews

### The Need and Feasibility

Linguistically, the world is very diverse: the languages spoken by most native speakers are Mandarin (14%), Spanish (6%), and English (5%) [2]. Although most educated health professionals and researchers can read texts in English, many others are not able to do so. The Cochrane Collaboration's target audience is broad, including health professionals, consumers, caregivers, and policy makers. For many of these groups, the proportion of people who can benefit from research-related information limited to English is actually quite small. In any country in the world, information about the effectiveness or harm of an intervention should ideally be available in the language used by its population, thus increasing the chances that this information is consulted. As an example, consider Africa, the continent with the greatest disease burden. In addition to multiple local languages, 115 million people across 31 countries speak French [3]. Thus, Cochrane Reviews made available only in English severely limit their potential to inform decision-making where evidence about the benefits and harms of healthcare interventions is needed urgently [4].

It seems clear that translation is a crucial strategy to meeting that challenge and, in fact, The Cochrane Collaboration's principles include the explicit commitment to enable wide participation by reducing barriers to contributing, encouraging diversity, and promoting access to its outputs through wide dissemination to meet the needs of users worldwide [5]. Fortunately, diverse initiatives have been promoted within The Cochrane Collaboration that permit the organization to be optimistic about the achievement of those goals. Translations have the potential to increase the usage of Cochrane Reviews. *La Biblioteca Cochrane Plus* (www.bibliotecacochrane.com), the Spanish version of *The Cochrane Library*, has pioneered translation and has become the most comprehensive project to translate Cochrane content (Box 1). Since 2003, its usage statistics have consistently demonstrated that universal access to content in Spanish across Spain and Latin America is used by millions of people every year. French translations of all abstracts of Cochrane Reviews published since 2010 and of content on the consumer-focused Cochrane Summaries website (www.summaries.cochrane.org) have shown this effect as well. The number of visitors to the French-language version of Cochrane Summaries per month has almost quintupled, from about 10,000 in September 2012 to 50,000 in May 2013. Over the same period, France moved from ranking eighth to second among the countries with the highest rates of access of Cochrane Summaries.

**Box 1. Biblioteca Cochrane Plus**

La Biblioteca Cochrane Plus (www.bibliotecacochrane.com) is the Spanish version of The Cochrane Library. Designed and promoted by the Iberoamerican Cochrane Centre in 1998, it has become a reference electronic resource for the dissemination of Cochrane Reviews in Spanish-speaking countries. Free access has been funded by the Spanish Ministry of Health with contributions from the Pan American Health Organisation (via BIREME provision) for Latin American users. Until 2010 it included the full text of each Cochrane Review translated and updated when necessary. In the last 3 years and to keep the project sustainable, only the abstract, plain language summary, and most relevant parts of each Review are translated with a link to the full English version provided. In August 2013, full or partial content of 5467 Cochrane Reviews was available in Spanish. The average number of inquiries from users was around 3.5 to 4 million per year over the past 5 years and in 2012, more than 4.5 million people consulted the Biblioteca Cochrane Plus. This electronic resource also includes other evidence-based materials originally published in Spanish. Moreover, the Iberoamerican Cochrane Centre has also translated other evidence-based content including podcasts, training materials and guidance documents, such as the Cochrane Handbook for Systematic Reviews of Interventions, and the Methodological Expectations of Cochrane Intervention Reviews (MECIR).

Several other initiatives on a smaller scale than the Spanish and French translations have focused on the translation of selected abstracts and plain language summaries, often with non-professionals in mind as the primary audience. Such translations have been prepared or are planned in such diverse languages as Simplified and Traditional Chinese, Portuguese, Croatian, German, Japanese, Russian, Korean, and Indonesian (www.cochrane.org/editorial-and-publishing-policy-resource/translation-projects).

## Plans and Challenges

A number of challenges have emerged when translating Cochrane Reviews. The first is ensuring high quality across all translations, given the different methods and resources used for translating. Also, the meaning of certain terms and concepts may vary across cultures, even if they share the same language (Box 2).The second challenge arises as Cochrane Reviews are updated periodically, and keeping track of updates is a huge task. The third, specific to countries with several official languages, is the need to translate the same content into several languages concurrently (Boxes 3 and 4). The fourth is that available translations have been spread over different platforms, many of them partially outdated and difficult to track, in part because The Cochrane Collaboration has not previously developed a centrally co-ordinated and funded strategy. The fifth arises from trying to decide what content to translate: one option is to translate plain language summaries and abstracts of the most recent and up-to-date reviews. Translating the full text of all Cochrane Reviews may not be feasible in some settings, so user demand must be considered before engaging in costly and onerous translation activities. Some initiatives prioritize topics for translation by an assessment of decision-maker demand (Box 3). The final challenge is to address the need for communication between the different teams translating reviews, especially those using the same language. Given the way The Cochrane Collaboration is set up, it is in the ideal and natural position to streamline translation initiatives, ensure efficiency and prevent duplication of effort.

**Box 2. 19 Years of Small-Scale Translation of Cochrane Review Abstracts and Summaries in Evidence Update**

Since 1994, the Effective Health Care Research Consortium (EHCRC) has been active in many countries in preparing Cochrane Reviews and disseminating their findings, often through their own bespoke summaries called Evidence Update (www.evidence4health.org/evidence.htm). At various times, these summaries have been translated into Portuguese, Spanish, French, Thai, Russian, and Chinese. However, translation is usually not straightforward: in one institution in Thailand, a country with a strong tradition of bilingual medical training, users preferred the original English language product. In other countries, the accuracy of the translation was problematic, not because of poor translation, but because the words mean different things. For instance, work in China on translation in qualitative research showed that there are often phrases and words without any English equivalent or with more than one meaning [9]. In a recent pilot translation project at the Chongqing Medical University in China, 100 abstracts and plain language summaries of Cochrane Reviews were first translated from English to Traditional Chinese by a team of experienced translators from Taiwan and then converted into Simplified Chinese characters. However, machine translation in the second step resulted in versions that were virtually unusable, and the team had to go back to the English version and re-translate all of the abstracts. Quality assurance in the process was essential to obtain correct translations.

**Box 3. Cameroon Experience**

Cameroon is – apart from Canada – the only other bilingual country with both French and English as official languages. However, French is the predominant language. The Centre for Development of Best Practices in Health (CDBPH) in collaboration with the Effective Health Care Research Consortium (EHCRC) is working on a 5-year project to enhance the uptake and use of up-to-date health research evidence primarily from the CDSR. Efforts to reach non-English-speaking health stakeholders in Cameroon were hampered by language barriers, notably in building capacity for conducting Cochrane Reviews, reading and applying the evidence, or communicating it to policy makers. Consequently, significant resources were diverted to providing translations of Cochrane Review abstracts and plain language summaries. A list of priority reviews on topics relevant to Cameroonian stakeholders and EHCRC targets was established. The quality of these translations was verified by the French Cochrane Centre and Cameroonian teams. The CDBPH also started producing bilingual evidence assessments – summaries of Cochrane Reviews adapted to the local context. These translations have led to a higher uptake of Cochrane products, with more downloads from the CDBPH website (www.cdbph.org) and demands for other evidence products, such as Policy Briefs and Rapid Responses.

**Box 4. Dissemination in a Multilingual Country – The Experience in Switzerland**

Switzerland has about 8 million inhabitants; most of them speak German, French, or Italian. English proficiency is widespread among health professionals but many prefer reading educational material in their own language. The challenge for Cochrane Switzerland (swiss.cochrane.org) is to serve a multilingual health care community. The Cochrane Branch collaborates with one medical education journal in each language region (Revue Médicale Suisse, PRAXIS, and Tribuna Medica Ticinese). New or updated Cochrane Reviews of interest to general or internal medicine practitioners are continuously selected and summarised. To put them in context, clinicians are invited to write short clinical scenarios and questions, which are then answered using the evidence from the Cochrane Review. In this way, limited resources of the Cochrane Branch can be used effectively in order to reach multiple audiences.

Different methods have been used for translation projects (Table 1), with no formal evaluation of the respective outputs and efficiency. Until now, conventional translation by professionals or volunteers has predominated. However, even if limited to short texts such as abstracts, these translation methods represent considerable investment that are difficult to maintain over time and, due to the related cost, hard to extend to multiple languages. In fact, only those projects with permanent public funding have succeeded to maintain the necessary continuity.

**Table 1:** Methods for Translation of Cochrane Reviews and Related Content.

Method	Users	Details	Quality	Resource Implications
Professional translation and editing	Most larger Cochrane translation projects	Translations are contracted, with further editing by content experts.	High	Highest cost compared to the other models, thus least sustainable
Computer-aided translation (CAT; e.g., Déjà Vu)	Iberoamerican Cochrane Centre for *La Biblioteca Cochrane Plus*	Professional translators and editors are capable of using CAT software. Its most recent versions combine its output sequentially with machine translation.	High, especially when the software's translation memory expands continuously.	High cost, although the price is graded depending on the number of repetitions and matches with content in the memory. New technologies and software can facilitate coordination and reduce costs (e.g., linked data).
Machine translation (without further validation)	Not used	Use of automated software. Many free or paid-for online/desktop solutions exist.	Lowest compared to the other methods, but depending crucially on software's translation memory and complexity of original content.	Low cost but currently not reliable enough.
Machine translation with human validation	Not used yet, but being tested by QUARTET M (Box 5).	Use of automated software with further editing by skilled person.	Very good. Open software can be trained with existing material, especially if content is written in a standardised language (e.g., Simplified English).	Reasonable cost and sustainable. Expenses mainly from adapting software and translated material used for “training” software, if amount of translated content available is insufficient.
Machine translation with human validation by crowd-sourcing	Epistemonikos, a network created by the Iberoamerican Chilean Cochrane Node at the Pontificia University in Santiago (www.epistemonikos.org).	Crowd-sourcing in a social network, where everyone can contribute to translations as much or little as they like.	Likely to vary, but probably acceptable, as mostly committed people would contribute and correct each other (Wikipedia principle).	Very low cost (free software), although some co-ordination is needed to implement style guides, glossaries, and training activities.

CAT, computer-aided translation; QUARTET M (Qualité de l'Aide à la Rédaction et de la Traduction; Evaluation du Transfert d'information en Médecine), multidisciplinary research group including the French Cochrane Centre, the Laboratoire d'Informatique pour la Mécanique et les Sciences de l'Ingénieur (LIMSI-CNRS, Paris Sud University), and the Centre de Linguistique Inter-Langues, de Lexicologie, de Linguistique Anglaise et de Corpus (CLILLAC-ARP, Paris Diderot University).

Sustaining any translation effort depends on the extent to which certain technologies (such as machine translation) can support humans in the accurate translation of content from English into other languages. Relying exclusively on human translation is probably too variable and expensive. However, whether a translation technology performs well depends directly on the complexity of the language and the specificity of the terminology used. The language used in Cochrane Reviews is both specialised from a technical point of view (as it uses specific methodological terms) and diverse (as reviews are conducted in various fields of health care). Therefore, a first line of strategic development around translations is to develop a controlled language and then to use it as much as possible in order to standardise the text sources that are to be translated. The quality of machine translation improves significantly if the primary text uses terminology in an unambiguous and consistent way. A controlled language is defined as using a restricted vocabulary, streamlined grammar, and a defined set of stylistic rules [6]. Using a controlled English language to simplify technical texts with a view to improve the efficiency of future translations would also increase the readability of the source text for users who are less proficient in English [7]. Some problems faced with partial or full translation of Cochrane Reviews have been related to ambiguity of the language in the source documents. This feature is not surprising if one considers that the *CDSR* is compiled of text written by several thousand different authors, many of whom are non-native English speakers.

Conceptually, the approach of moving to a controlled language is close to one already developed by the Cochrane Effective Practice and Organisation of Care (EPOC) Group, one of the 53 Cochrane Review Groups, which helps Review authors formulate clear and consistent statements about the effect of interventions [8]. A defined list of qualitative statements is proposed to express the magnitude of effect and the quality or certainty of evidence; for instance, “The [intervention] probably slightly improves/reduces [outcome] for an intervention with a moderate quality of evidence and a less important benefit/harm.”

The Spanish and French translation projects have to date been working with computer-aided translation (CAT) software such as Dejà Vu. This software has the capacity to learn and recognise language patterns, which helps the translators to be consistent and reduces the amount and cost of text to be translated (Table 1). However, using CAT is expensive because it relies heavily on human input. As a result, it seems more promising to explore and improve the performance of automatic translation, assuming that it would be greatly facilitated if the original text in English is as standardised as possible. Based on these criteria, the French Cochrane Centre has partnered with language experts to train a machine translation system using large multilingual text sources and Cochrane Review abstracts already translated into several languages (Box 5).

**Box 5. The Potential of Using Simplified English**

Using Simplified English is increasingly recognised as an important strategy to facilitate translation in various fields. For instance, in aircraft manufacturing and maintenance, it was employed to render the technical documentation easier to use and thus safer, as well as more efficient. The French Cochrane Centre has formed a multidisciplinary research group (QUARTET M) with the Laboratoire d'Informatique pour la Mécanique et les Sciences de l'Ingénieur (LIMSI-CNRS, Paris Sud University), one of the country's largest research groups working on language technologies, machine translation, and statistical language modelling, and with the Centre de Linguistique Inter-Langues, de Lexicologie, de Linguistique Anglaise et de Corpus (CLILLAC-ARP, Paris Diderot University) specialized in phraseological and terminological analysis, technical writing, and the development of writing aid tools for Scientific English. The main focus of the project is to train a machine translation system using large multilingual text sources and Cochrane Review abstracts already translated into several languages. First results from tests using a sample of 600 abstracts translated into French to train the memory of the software have been promising. Rather than focusing on the technical problems of automatic translation only, QUARTET M has included a novel approach to investigate whether the adoption of Simplified English and writing aid tools has the potential to increase translation productivity, inclusiveness, accessibility, readability, and user experience. Most importantly, it is assumed that it would increase the feasibility of machine translation with human validation considerably. Besides, the usage of Simplified English could improve identification of Cochrane content in Google searches and enhance the development of derivative products by facilitating automatic extraction of data.

The performance of any software used for translation increases with the amount of suitable training text that is fed into it. A body of literature, glossaries of technical terms, or systematic domain terminologies already translated in several languages can be used to improve its performance in a certain area of content and to reduce the workload related to post-editing. For instance, international organisations, such as the World Health Organization or the European Medicines Agency, have large multilingual resources relevant to health care. Also, the CONSORT Statement and other reporting guidelines contain relevant methodological terminology and are available in several languages (www.equator-network.org/). The amount and quality of the material available in different languages varies greatly and therefore limits its usability to improve machine translation. Research may help to identify what minimum size of corpora is required to achieve the desired level of quality.

Another interesting approach to translation, taken by Epistemonikos (www.epistemonikos.org), is based on crowd-sourcing. The project uses freely available software to translate medical texts, including Cochrane Review abstracts, into Spanish (among eight other languages). The translated texts are further reviewed by volunteering clinicians or senior students without formal training in translation, who receive continuous feedback from more experienced contributors (Table 1). Tagged key terms are stored as linked data, which make them searchable, e.g., in taxonomic or Patient-Intervention-Comparison-Outcome (PICO) searches in other products and software, and useful for future developments. Regardless of the software used for translation, validating the text produced by the machine is always necessary. Again, a collaborative approach such as that taken by Epistemonikos can be efficient and valuable in terms of social contributions, similar to Wikipedia.

A strategic approach to producing content in languages other than English could encompass The Cochrane Collaboration's training materials or derivatives, and perhaps even its content management applications. For example, a researcher could conduct a systematic review in his or her own language and have it translated into English at a later stage. Also, a writing aid tool could be integrated to facilitate the authoring of reviews in controlled or simplified English. Such a tool would automatically propose specific standardised expressions or paragraphs to assist review authors in writing in an unambiguous way, thus simplifying the translation and improving the readability for both non-native and native English speakers.

## Providing Access

The best option for providing access to translated Cochrane Reviews may be to publish all available translations in any language, including English, on a common multilingual platform. The interface should allow for user-friendly search and browse in all available languages. The design should facilitate quick navigation between the different languages (again, taking the Wikipedia model). In terms of search, there is a need to distinguish between two different approaches for returning search results by language: the more simple is a search and browse function for various languages that only returns results for one language at a time; and the more complex (and probably most challenging to implement) is a multilingual search engine, which returns results in multiple languages at the same time. Another important aspect to take into account is mobile access via applications (apps). The free iPad application for *The Cochrane Library* launched in 2012 has proved popular across the globe. Promoting use of small-screen devices and applications that free up how and when people can access Cochrane content in different languages will be an area of further development. It will be critical especially in low-resource countries where smartphones are usually more accessible than computers with reliable internet connection.

In 2013, The Cochrane Collaboration took the first step towards its vision of making all Cochrane Reviews open access: all Cochrane Reviews published from 1 February 2013 will be free to view 12 months after publication (green open access) or immediately if authors choose to pay a publication fee (gold open access). Other routes to promote access to *The Cochrane Library* include providing free one-click access to all people in low- and middle-income countries included in the World Health Organization's Health InterNetwork Access to Research Initiative (HINARI) (www.thecochranelibrary.com/view/0/FreeAccess.html). Historically, public funds have sustained translation initiatives and, accordingly, access to the translated content exceeding the abstract and plain language summaries has been free to view in the respective countries. One example is *La Biblioteca Cochrane Plus*, which is free to view in Spain as well as in eligible HINARI countries in Latin America. All translated plain language summaries and abstracts in the *CDSR* are free to view anywhere. Over the coming months and years, The Cochrane Collaboration will be working with funders and its publisher to explore how to move to a publishing model that is based upon open access but also ensures its economic sustainability. Including translations in these discussions as well as establishing sound permanent alliances in different geographical regions will be crucial to developing sustainable translation projects.

## Conclusions

As the world's largest producer of systematic reviews, The Cochrane Collaboration sets at its 20^th^ anniversary a high priority on its global reach and relevance by engaging in the production of content in as many languages as possible. The success of previous experience demonstrates that when texts in local languages are provided, usage increases substantially. At the same time, the potential to produce cost-effective translations of good quality using new technologies, crowd-sourcing, or a combination has never been greater. If this approach is accompanied by efforts aimed at simplifying and making the use of English in reviews more consistent, there is a real prospect that Cochrane Reviews can become a genuinely international resource. As a global network, The Cochrane Collaboration has a considerable technical capability and benefits from two decades of work experience in diverse settings and languages. The organization will need, however, to continue to build partnerships around complementary expertise and with funders to ensure that people can access the information they need for healthcare decisions in languages of their choosing.

## Supporting Information

Table S1 **Comparison of expert reviews, systematic reviews, and Cochrane Reviews.**
(DOCX)

## Abbreviations

CAT: computer-aided translation
CDBHP: Centre for Development of Best Practices in Health
CDSR: Cochrane Database of Systematic Reviews
EHCRC: Effective Health Care Research Consortium
HINARI: World Health Organization's Health InterNetwork Access to Research Initiative
MECIR: Methodological Expectations of Cochrane Intervention Reviews
PICO: Patient-Intervention-Comparison-Outcome
QUARTET M: Qualité de l'Aide à la Rédaction et de la Traduction
Evaluation du Transfert d'information en Médecine
