# Supplementary material for: Translating Cochrane Reviews to Ensure that Healthcare Decision-Making is Informed by High-Quality Research Evidence
Source: PLoS Med. 2013 Sep 17;10(9):e1001516. doi: 10.1371/journal.pmed.1001516 (PMC3775718; doi:10.1371/journal.pmed.1001516)
Supplement: Table S1 — Comparison of expert reviews, systematic reviews, and Cochrane Reviews. (DOCX) [file pmed.1001516.s001.docx]

**Table S1. Comparison of expert reviews, systematic reviews, and Cochrane Reviews.**

| Aspect | Expert review^1^ | Reliable systematic review ^2^ | Unique to Cochrane Reviews |
| --- | --- | --- | --- |
| Question | Usually starts with general discussion | Starts with clear question or hypothesis | Required and PICO discussed with editors |
| Protocol | None | Completed before starting | Refereed and published |
| Methods | Vary and often not specified | Clearly defined methods | Standardised, and written by leading methodologists working collaboratively.  Access to specific methods advice provided out of good will |
| Authors | Usually dominated by topic experts | Team includes methodologists | Team includes methodologists and topic experts, and sometimes consumers |
| Conflicts of interest | Generally poorly managed | Managed variably | Adhere to published conflicts of interest policy^4^ |
| Selecting studies to include | Inclusion criteria usually vague; does not describe why studies are excluded | Explicit inclusion criteria | Explicit inclusion criteria in the protocol and studies excluded and the reasons provided in the final review |
| Literature searches | Usually not attempted | Strive to locate all relevant published (generally only in English-language journals) and unpublished data | Methods standardized  throughout The Cochrane Collaboration, which include searching trials published in other languages and registered in a central database (CENTRAL) |
| Assessing risk of bias in studies | Usually does not consider differences in study methods or risk of bias | Investigation of risk of bias and sources of heterogeneity | Systematic investigation of risk of bias and sources of heterogeneity is pre-specified and rigorously applied |
| Data synthesis | Often does not differentiate between methodologically sound and unsound studies | Conclusions based only on methodologically sound studies | Conclusions based only on methodologically sound studies with routine sensitivity analysis |
| Formal appraisal of strength of evidence | Not done | Usually not done | Some new Reviews use GRADE methods and all new Reviews should use GRADE criteria to discuss results |
| Peer review | By content experts | Not always refereed by people familiar with systematic reviews | Includes peer review by systematic review specialists and often also consumers |
| Updates | Usually not available | None | Reviews in current topics are kept updated^3^ |
| Avoiding duplication | No mechanism to avoid duplication | No mechanism to avoid duplication | Cochrane mechanisms to avoid duplicate reviews.  Updating also avoids waste. |

^1^ Sometimes called “narrative review”, “viewpoint”, “opinion”, “thorough review done by experts”.

^2^ The Cochrane Collaboration has established methods and software that are widely available.

^3^ From 2014, editorial teams will be able to categorise reviews that no longer need updating so that preferably reviews of current questions, where equipoise or uncertainty remains, will be updated.

^4^<http://www.cochrane.org/editorial-and-publishing-policy-resource/conflicts-interest-and-cochrane-reviews>
